# Particle Surface Softening as Universal Behaviour during Flash Sintering of Oxide Nano-Powders

**DOI:** 10.3390/ma10020179

**Published:** 2017-02-14

**Authors:** Rachman Chaim

**Affiliations:** Department of Materials Science and Engineering, Technion—Israel Institute of Technology, Haifa 32000, Israel; rchaim@technion.ac.il; Tel.: +972-4-829-4589

**Keywords:** flash sintering, spark plasma sintering, densification, plasma, electric field, invasive percolation, nano-powders, oxides

## Abstract

The dissipated electric power in oxide powder compacts, subjected to flash sintering, is several hundreds of W·cm^−3^. This power is analyzed considering local softening/melting and transient plasma/liquid formation at the particle contacts due to thermal runaway. The sudden increase in compact electric conductivity and dissipated power referred to current percolation through the softening/liquid formed at the particle contacts, at the percolation threshold. The energy-balance and heat transfer considerations during the transient flash event are consistent with the local heating of the nanoparticle contacts to the ceramic melting temperature, or above it. The formation of the plasma by field emission of electrons is also considered.

## 1. Introduction

Flash sintering is a novel technique by which ceramic powder compact is densified during a few seconds under simultaneous furnace heating and an applied electric field. The sudden densification is accompanied by an optical flash [1,2], hence ‘flash sintering’, although optical flash is claimed to also be observed in dense polycrystalline specimens and single crystals. Nevertheless, no experimental evidence is reported for the latter claims. The rapid densification often takes place at certain temperature-electric field values; higher fields need lower flash onset temperatures [3]. One interesting aspect of the process is that the dissipated electric power per unit volume at the flash event ranges over a few hundreds of W·cm^−3^ [4], irrespective of the oxide composition. Recently, Raj [4] analyzed this universal behaviour by analysis of the Joule heating. However, his model assumed pure solid-state sintering, without temperature gradients within the powder compact. The conclusion from the above model was that “Joule heating is a necessary but not a sufficient condition for flash sintering” [4]. 

Several attempts were made to model the flash sintering based on the thermal and electrical energy balance of the black body, where Joule heating, thermal convection, conduction, and radiation are considered [4,5,6,7,8]. Most of the models assume solid-state sintering with constant body temperature. However, the energy balance by a dynamic model, assuming non-uniform temperature, pointed to temperature gradients up to several thousand degrees along the cylindrical specimen diameter [6], although much lower temperature gradients of ~100 °C were experimentally reported [9]. Although these models relate the flash event to thermal runaway, none of them considered local melting or surface softening at the particle surfaces. The dissipated power of several hundreds of W·cm^−3^ originates from the artificial control of the voltage and its exchange to the current control mode, once the flash occurs. In principle, a continuous constant voltage mode may lead to the same results of rapid powder densification, albeit with possibilities of local electric breakdown and specimen disintegration. In this respect, Park and Chen [10] used 8YSZ (8 mol % Yttria Stabilized Zirconia) specimens with different cross-sections, and measured specimen temperatures as high as 2500 °C, very close to the specimen melting point. They related the high electric resistance of the post flash sintered specimens to the extreme heat and thermal shock damage [10]. Therefore, the artificial exchange of voltage to current control mode, after the flash event started, does not negate the probability for particle surface melting/softening in the absence of such a mode exchange. Analysis of the observed luminescence wavelength at 1175 nm from the flash of 3YSZ (3 mol % Yttria Stabilized Zirconia), via Planck’s law for black body radiation (BBR), revealed temperatures as high as 2360 °C, compared to the highest measured specimen temperature of 1690 °C [11]. Nevertheless, deconvolution of the non-analyzed overlapping peak around ~900 nm wavelength (Figure 5a in ref. [11]) by the same analysis may result in temperatures up to ~3080 °C. Analysis of the emission peaks assigned at 736 nm and 625 nm from the flash sintering of SrTiO_3_ (Figure 4 in ref. [12]) reveals temperatures as high as 3768 °C and 4437 °C, respectively. A finite element model was used to calculate the temperature profile within the 8YSZ specimen subjected to 5 s of flash [13]. Internal temperatures higher than 2100 °C were calculated compared to the maximum surface temperature of 1015 °C; the actual specimen temperature measured by the thermocouple was 863 °C. Therefore, photoemission from the flash exhibits very high temperatures that are involved in the sintering process, although the validity of BBR for temperature evaluation during the flash sintering is still controversial. 

Shomrat et al. [8] analyzed the energy balance for flash sintering of SrTiO_3_ within a dilatometer using a finite differences approximation. The Joule heating and radiation were the main energy components immediate to and at the flash event; their corresponding values were higher by one to two orders of magnitude than the energies associated with convection or conduction. This is not surprising at high temperatures, due to the power-law temperature-dependence of the radiation, compared to the linear relation in convection and conduction. This approximation was also adopted by Dong and Chen [7] who derived an expression for the flash onset temperature by equating the Joule heating and the radiated energy. They showed the vertical nature of the Joule heating above the flash onset temperature, albeit this power within a granular system is due to the percolating current, hence, it increases in a power-law regime. These authors also showed the Joule heating to be solely responsible for the thermal runaway [14]. Consequently, the accumulated excess power generated by Joule heating during the few seconds beyond the flash onset temperature is very valuable for determining the local temperature raise within the specimen. Although this transient stage is not in equilibrium, its heat transfer conditions are quasi-stationary (see below), and therefore justify the present analysis. The goal of the following analysis is to show that the correct energy balance at the flash event can be obtained while taking into account partial melting of the particle surfaces, due to the local thermal runaway at the particle contacts.

## 2. Analysis and Discussion

### 2.1. Energy Balance during the Flash Event

Recently, an invasive percolating model was used to relate the rapid densification during flash sintering to the attractive capillary forces formed at the particle contacts, due to their local melting [15]. The percolative nature of flash sintering is confirmed due to the electrochemical character of the process [16,17,18], the asymmetric flash ‘ignition’ at one end of the specimen [1,2], as well as different microstructures formed at the cathode and anode [18], and the formation of dense and porous ‘islands’ at interrupted flash experiments [16]. It is interesting to evaluate the flash sintering process energetics with respect to a possible transient process of partial softening/melting of the particle surfaces at the percolation threshold. The question is whether the excess input power suffices for partial surface melting at the percolation threshold to form a transient liquid, in addition to heating the specimen to its average surface temperature (*T_surf_*) as measured by the pyrometer, or by other means. 

Following the previous models developed for flash sintering (i.e., [4,5,6,7,8]), the excess between the powers generated by Joule heating and dissipated by radiation and convection, is the source for the temperature increase within the specimen. This excess power per specimen unit volume can be fairly well expressed by:

(1) $$\Delta Q_{excess}=\frac{V^{2}}{R_{s}\left(T\right)}-h\frac{A_{s}}{V_{s}}\left(T_{surf}-T_{fur}\right)-\frac{A_{s}}{V_{s}}\varepsilon_{em}\sigma_{SB}\left(T_{surf}^{4}-T_{fur}^{4}\right)$$

where *V* is the applied voltage per unit thickness, *R_s_*(*T*) is the temperature-dependent electric resistance of the specimen, *A_s_* is the surface area for convection/radiation, *V_s_* is the specimen volume, *h* is the convection coefficient, *ε_em_* is the emissivity, *σ_SB_* is the Stefan-Boltzmann constant for black body radiation, and *T_surf_* and *T_fur_* are the specimen (free) surface temperature and furnace temperature, respectively.

Since the numerical solution and the calculated values have important consequences for the model validity, we took into consideration all the heat components, even though some of them might be negligible.

Assuming the same excess power per unit volume spent for heating the specimen and partial melting/softening of the particle surfaces (at the contact points) that fulfils the percolation threshold for the current through the liquid component with lower electric resistance, leads to:

(2) $$\Delta Q_{excess}=\frac{\rho_{g}\cdot \rho_{0}C_{p}}{\Delta t}\left(T_{surf}-T_{fur}\right)+T_{cont}\cdot \frac{\Delta S_{m}}{W_{mol}}\cdot f_{per}\frac{\rho_{g}\cdot \rho_{0}V_{s}}{\Delta t}$$

where *ρ_g_* is the relative green density, *ρ*_0_ is the theoretical density, *c_p_* is heat capacity, Δ*t* is the flash event duration in the voltage control mode, *T_cont_* is the temperature at the particle contacts, $\Delta S_{m}$ is the entropy of melting, *W_mol_* is the molar weight, and $f_{per}$ is the current percolation threshold. The current percolation threshold is the critical volume fraction of the ‘conducting’ medium, which is needed to provide a continuous path for the electric current percolation through the material between the two electrodes.

The first term on the right hand side of Equation (2) represents the energy input to increase the temperature of the solid particles; its corresponding temperature *T_surf_* is measured as the surface temperature of the specimen (no particle contacts). However, the second term on the right hand side of Equation (2) represents the temperature increase to *T_cont_*, at the particle contact loci, where softening/melting takes place. This second term originates from Δ*H_m_ = T_m_·*Δ*S_m_* at melting. However, in order to verify whether *T_cont_* reaches the melting point, we left it as variable *T_cont_*, and its final value will be determined from the energy balance; if the resultant *T_cont_* ≥ *T_m_*, (*T_m_* is the melting point) then the present analysis is correct, and melting or higher temperatures are achieved at the particle contacts. The corresponding volume of the melted particle contacts is the product of the percolation threshold, *f_per_*, and the specimen volume. In addition, we emphasize that *T_cont_* does not represent the *specimen* surface temperature, *T_surf_*, at the flash event, since the external surfaces of the specimen are free of particle contacts.

Rearrangement of Equation (2) yields the temperature at the particle contacts as:

(3) $$T_{cont}=\frac{1}{\Delta S_{m}\cdot f_{per}}\left[\frac{\Delta Q_{excess}\cdot \Delta t\cdot W_{mol}}{\rho_{g}\cdot \rho_{0}}-c_{p}\left(T_{surf}-T_{fur}\right)\right]$$

Since we use the entropy of melting (fusion), Equations (2) and (3) are valid providing that the calculated temperature at the particle contacts is equal to or higher than the melting point of the oxide (i.e., *T_cont_* ≥ *T_melt_*), as mentioned above. The first term on the right hand-side of Equation (1) represents the Joule heating. However, flash sintering at the flash event is a dynamic process, during which the material properties may change. Exchange of the voltage to current control mode during the flash event (the so-called stage I to stage II), leaves a power peak, the height of which is the maximum input power. The overall power peak was previously approximated by a triangle shape spike with a width at half maximum of 1 s [19,20]. Here we calculate the excess power generated at the voltage control mode only, from the flash start (onset temperature) to the maximum of the power peak. Typical durations of the total flash event peak range between 3 to 10 s [8,19,20]. For consistency, and following Raj [20], we used published experimental power data during the flash event, at constant voltage, as Joule heating [19], whenever the flash event duration was provided. We calculated the Joule heat using the area of a sharp right-angle triangle spike (i.e., the first half of the triangular spike) the height of which is the peak maximum (*Q_peak_*), and its abscissa is the actual flash event duration at the constant voltage regime (only), according to:

(4) $$Q_{Joule}=\frac{Q_{peak}\;\cdot \;\Delta t}{2}$$

All the parameters in Equations (1)–(4) are often measured or known during flash sintering, except *T_cont_* at the particle contacts. Calculating the Joule heating via Equation (4), using the peak area and its corresponding time *Δt*, and substituting it into Equations (1) and (3), we estimated the temperature at the particle contact points, *T_cont_*. If the calculated temperature at the particle contacts is equal to or higher than the melting point of the oxide (i.e., *T_cont_* ≥ *T_melt_*), then the solution is valid and may indicate local melting at the particle contacts.

For simplicity, we used the properties of the dense materials when their measured values were absent, and the correct material density where needed. This trend leads to a more conservative treatment with respect to the contact temperature of interest (results in lower values). We used the following values: emissivity of 0.9 for oxides [4], the Stefan-Boltzmann constant for black body radiation of 5.67 × 10^−8^ W·m^−2^·K^−4^, and the percolation threshold of 0.247 for the invasive percolation model [21]. In addition, we emphasize that *T_cont_* does not represent the *specimen* surface temperature, *T_surf_*, at the flash event, since the external surfaces of the specimen are free of particle contacts. The measured surface temperatures in Table 1 (the end of the 1st stage) were used for *T_surf_* in Equation (1).

Using the measured (not calculated) published data for 3YSZ [19,20] and SrTiO_3_ [8], where flash event durations were clearly indicated, their properties [22,23,24,25,26] and flash sintering conditions, together with their corresponding calculated temperature, *T_cont_*, are listed in Table 1. In both oxides, the temperatures at the particle contacts are far above their corresponding melting points, i.e., 3114 °C versus 2680 °C for 3YSZ, and 4216 °C versus 2080 °C for SrTiO_3_, and confirm the possibility for local melting/surface softening at these loci. These calculated temperatures are in agreement with those calculated from the flash photoemissions mentioned above [11,12]. Numerical simulations by Holland and coworkers [27] on the local joule heating during field assisted sintering of ionic ceramics revealed significant temperature gradients between the particle’s core and the contact point at its surface. These calculations further support the existence of temperature gradients along the particle radius. Therefore, the energy balance during the transient stage of the flash event may be achieved by particle melting/softening of the particle surfaces at their contact points, subjected to thermal runaway.

### 2.2. Possibility for Plasma Formation

The very high temperatures calculated above are higher than the melting points; they may reveal the formation of atmospheric-pressure plasma, due to the electron release from the charged particle surfaces subjected to locally high electric fields, and ionization of the surrounding air/atmosphere. The atmospheric-pressure plasma due to the breakdown of air, according to Paschen’s law, can form at 340 volt and 550 volt for parallel inter-particle gaps of 9.2 μm and 2.6 μm, respectively [28]. In such a plasma, formed by the gas breakdown, both temperatures of the excited electrons and gas ions merge to values close to 6000 K at atmospheric pressure (760 Torr). Nevertheless, controlled breakdown experiments in air with electrode separations from 400 nm to 45 μm showed that Paschen’s law is not valid for separations below 10 μm [29,30]; breakdown voltages are either equal to the Paschen minimum [30], or decrease with the decrease in distance between the electrodes [29]. Therefore, the theory of avalanche electrons accelerated by the local electric field, which causes the ionization of the surrounding air molecules (Paschen’s law), does not hold for narrow micrometer and nanometer size separations, which characterize the ceramic powder compacts. This plasma at the sub-micrometer and nanometer separations (i.e., pore size) is explained by the edge-type breakdown, where the sparks select an energetically favorable distance, due to the electrode (particle) shape and curvature (radius) [31], with the dominant effect of field emission of the electrons [32]. A previous analytical model, which was developed for the plasma formation during the spark plasma sintering [33], and was verified experimentally to cause particle surface melting [34,35], may be applicable for the flash sintering. Numerical simulations of the local field strength during the early stage of field assisted sintering in dielectric materials exhibited the buildup of high local fields at the particle contacts, which may explain the propensity for plasma formation at these loci [36]. In addition, flash sintering of α-alumina with different levels of pre-sintering was analyzed and referred to Frenkel’s ‘pre-breakdown’ behavior in the presence of high local electric fields [37]. 

### 2.3. The Heat Transfer Regime

An important question is whether the high temperature gradient formed between the solid particle, *T_surf_*, and its melted surface, *T_cont_*, can lead to immediate temperature homogenization, and prevent the contact from overheating. Lebrun et al. [9] estimated typical thermal diffusivity for the present 3YSZ as 7.4 × 10^−7^ m^2^·s^−1^. Therefore, the thermal relaxation time for temperature homogenization across a nanoparticle of 33 nm radius is ~1.5 ns, compared to the 1 s duration of the flash event; hence, thermal homogenization is an immediate process. On the other hand, these times also provide conditions for quasi-stationary heat transfer from the nanoparticle to its surroundings, by convection and radiation. A similar behavior is expected for the SrTiO_3_ powder.

The problems of heat transfer within and from hot nanoparticles was thoroughly treated, especially using laser-induced incandescence-based diagnostic techniques [38,39,40,41]. For the case of stationary nanoparticles in the gas medium heated by a laser (either nano-second pulses or continuous irradiation), several heat conduction and mass transfer regimes were observed. The heat transfer regime in such systems depends on the Knudsen number (*Kn*), which is expressed as a ratio between the mean free path (*λ_MFP_*) of the gas molecules surrounding the particle at the temperature and pressure of interest, and the characteristic length scale of the particle (i.e., particle radius, *r*) [40]:

(5) $$Kn=\frac{\lambda_{MFP}}{r}$$

The value *λ_MFP_* is temperature dependent, and expressed by:

(6) $$\lambda_{MFP}=\frac{\mu }{p}\sqrt{\frac{\pi k_{B}T}{2m}}$$

where *μ* is the gas viscosity, *m* is the gas molecular mass, *k_B_* is the Boltzmann’s constant, and *p* and *T* are the gas pressure and temperature, respectively.

The quasi-stationary heat transfer from the hot nanoparticle to its surrounding gas may take place at three different regimes, depending on its Knudsen number. At sufficiently small Knudsen numbers (*Kn* ≤ 0.01), heat transfer occurs by the continuum regime. At sufficiently large Knudsen numbers (*Kn* ≥ 10) the free-molecular regime dominates, whereas at intermediate Knudsen values (0.01 ≤ *Kn* ≤ 10), the conduction is in the transition regime (composed of both continuum and free-molecular behaviors, and interpolates between the two). Heat transfer in the continuum regime is diffusion-controlled and depends on the gas temperature distribution near the particle. In contrast, the physics of the free-molecular regime is controlled by the collisionless (Langmuir) layer surrounding the particle, which causes a temperature jump within the limiting sphere; the sphere layer thickness is of the order of *λ_MFP_*. Similar physical effects are observed by the molecular dynamic simulation of heated solid nanoparticles up to melting, while the surrounding liquid did not boil [42]. The surface temperatures at the limiting sphere may be as high as three times the gas/liquid temperature far from the particle.

The corresponding value of *λ_MFP_* for air at 1 atm pressure and at room temperature (300 K) is 68 nm; its value increases to 580 nm at 1700 K [40]. In the following discussion, we will refer to the Knudsen numbers between the room temperature and the maximum furnace temperatures measured during the flash experiments (denoted as T_fur_ in our treatment). Using the mean free path calculated for air in 1 atm pressure versus temperature (Figure 4 in ref. [40]), the corresponding Knudsen numbers for the present 3YSZ nanoparticles (*r* = 33 nm) vary between 2 and 8.18 at 25 °C and 877 °C, respectively. The corresponding values for the SrTiO_3_ micrometer particles (*r* = 500 nm) vary between 0.136 and 0.50 at 25 °C and 815 °C, respectively. These Knudsen values, overall, fall into the transition regime range, which point to much slower heat transfer by convection within the distance, comparable to *λ_MFP_* from the particle surfaces. These Knudsen values are tangent to the free molecular heat transport regime, and support local increase of the temperature immediate to the particle surface, due to the presence of the collisionless layer surrounding the particles. Therefore, the smaller the particle, the higher the temperature established at its surface, at given flash process parameters; consequently, at a given temperature, a lower voltage is needed to initiate the flash event. Alternately, at a given voltage, a lower temperature is needed to initiate the flash event, consistent with the particle size effect observed during flash sintering [43].

Therefore, Joule heating preferably formed at the particle contacts, and dissipated first within the particles. However, prior to the particle melting, the accumulated heat should also conduct away from the particle surfaces into the gas/adjacent contacting particles. Due to the heat transfer in the transition regime, each nanoparticle contains a sphere of high temperature gas at its surface, the thickness of which is lower than *λ_MFP_*. Consequently, the adjacent particles must absorb the input energy, due to their symmetrical position with respect to their contact plan. In addition, the thickness of the limiting sphere of the molecular-free region increases with the temperature increase, and overlaps the growing number of the neighbor particles, as long as they are not sintered. This scenario is very close to the formation of a transient plasma plume at the flash event, consistent with visual observations up to now.

## 3. Summary and Conclusions

As was previously suggested [15,44] and experimentally reported [2,5,44], thermal runaway in the powder compact, which is subjected to flash sintering, may lead to local melting at the particle contact points. The present energy balance during the transient flash event confirms that particle surfaces subjected to thermal runaway may partially melt/soften at their contact points, and wet the particle to provide a preferred pass for current percolation. The densification of the powder compact takes place by local rearrangement of the particles, due to attractive capillary forces of the melt and/or enhanced surface diffusion. The invasive nature of the melt/softened surface at the contacts leads to an advancing front, which forms the percolative pass for the current flow through the liquid ‘circuit’. Once the percolative pass formed, densification takes place within fractions of a second. Careful observation of the snapshots from the flash event confirms its ‘ignition’ at one end (electrode) of the specimen prior to its propagation, in agreement with the percolation theory. Further confirmation of the flash sintering as a percolative system is due to the electrochemical character of the flash sintering process with an asymmetric nature. Considering the very high temperatures calculated above for the particle contacts, the formation of plasma also seems to be a plausible reason for the immediate powder shrinkage, due to particle surface softening; this may explain the visible optical flash observed during the short flash sintering events.

## Figures and Tables

**Table 1:** Materials and process parameters for two flash sintered oxide systems.

Parameter	(Units)	SrTiO_3_ [8]	3YSZ [19,20]
*ρ*	g·cm^−3^	5.11	6.05
W mol	g·mol^−1^	183.52	123.22
*ρ* green	%	60	52.5
A_s_	cm^2^	1.20	1.57
V_s_	cm^3^	0.09	0.196
A_s_/V_s_	cm^−1^	13.33	8.00
P applied pressure	MPa	dilatometer	sinter-forge @ 5 MPa
V	Volt·cm^−1^	600	100
c_p_ at °C	J·mol^−1^·K^−1^	126 at 886 K [22]	85 at 1000 K [23]
h	W·m^−2^·K^−1^	20 #	20 #
k	W·m·K^−1^	11.2	2.8 [24]
Δ*t* flash event	s	2.75	2.5
T surface	°C	886	1000
T furnace	°C	815	877
T melting	°C	2080	2680
ΔS melting (fusion)	J·mol^−1^·K^−1^	53.20 [25]	26.77 [26]
T particle contact	K (°C)	4489 (4216)	3387 (3114)

# For natural convection.

## References

[1] Jiang T., Wang Z., Zhang J., Hao X., Rooney D., Liu Y., Sun W., Qiao J., Sun K. (2015). Understanding the flash sintering of rare-earth-doped ceria for solid oxide fuel cell. J. Am. Ceram. Soc..

[2] Corapcioglu G., Gulgun M.A., Kisslinger K., Sturm S., Jha S.K., Raj R. (2016). Microstructure and microchemistry of flash sintered K_0.5_Na_0.5_NbO_3_. J. Ceram. Soc. Jpn..

[3] Downs J.A., Sglavo V.M. (2013). Electric field assisted sintering of cubic zirconia at 390 °C. J. Am. Ceram. Soc..

[4] Raj R. (2016). Analysis of the power density at the onset of flash sintering. J. Am. Ceram. Soc..

[5] Zhang Y., Jung J.I., Luo J. (2015). Thermal runaway, flash sintering and asymmetrical microstructural development of ZnO and ZnO-Bi_2_O_3_ under direct currents. Acta Mater..

[6] Todd R.I., Zapata-Solvas E., Bonilla R.S., Sneddon T., Wilshaw P.R. (2015). Electrical characteristics of flash sintering: Thermal runaway of Joule heating. J. Eur. Ceram. Soc..

[7] Dong Y., Chen I.W. (2015). Onset criterion for flash sintering. J. Am. Ceram. Soc..

[8] Shomrat N., Baltianski S., Dor E., Tsur Y. (2017). The influence of doping on flash sintering conditions in SrTi_1-x_Fe_x_O_3-δ_. J. Eur. Ceram. Soc..

[9] Lebrun J.M., Jha S.K., McCormack S.J., Kriven W.M., Raj R. (2016). Broadening of diffraction peak widths and temperature nonuniformity during flash experiments. J. Am. Ceram. Soc..

[10] Park J., Chen I.W. (2013). In situ thermometry measuring temperature flashes exceeding 1700 °C in 8 mol % Y_2_O_3_-stabilized zirconia under constant-voltage heating. J. Am. Ceram. Soc..

[11] Terauds K., Lebrun J.M., Lee H.H., Jeon T.Y., Lee S.H., Je J.H., Raj R. (2015). Electroluminescence and the measurement of temperature during stage III of flash experiments. J. Eur. Ceram. Soc..

[12] Naik K., Jha S.K., Raj R. (2016). Correlation between conductivity, electroluminescence and flash sintering. Scr. Mater..

[13] Pereira da Silva J.G., Lebrun J.M., Al-Qureshi H.A., Janssen R., Raj R. (2015). Temperature distributions during flash sintering of 8% Yttria-stabilized zirconia. J. Am. Ceram. Soc..

[14] Dong Y., Chen I.W. (2015). Predicting the onset of flash sintering. J. Am. Ceram. Soc..

[15] Chaim R. (2016). Liquid film capillary mechanism for densification of ceramic powders during flash sintering. Materials.

[16] Caliman L.B., Bouchet R., Gouvea D., Soudant P., Steil M.C. (2016). Flash sintering of ionic conductors: The need of a reversible electrochemical reaction. J. Eur. Ceram. Soc..

[17] Biesuz M., Sglavo V.M. (2016). Flash sintering of alumina: Effect of different operating conditions on densification. J. Eur. Ceram. Soc..

[18] Qin W., Majidi H., Yun J., van Benthem K. (2016). Electrode effects on microstructure formation during flash sintering of yttrium-stabilized zirconia. J. Am. Ceram. Soc..

[19] Francis J.S.C., Raj R. (2012). Flash-sinterforging of nanograin zirconia: Field assisted sintering and superplasticity. J. Am. Ceram. Soc..

[20] Raj R. (2012). Joule heating during flash-sintering. J. Eur. Ceram. Soc..

[21] Straley J.P. (1977). Critical exponents for the conductivity of random resistor lattices. Phys. Rev. B.

[22] Jacob K.T., Rajitha G. (2011). Thermodynamic properties of strontium titanates: Sr_2_TiO_4_, Sr_3_Ti_2_O_7_, Sr_4_Ti_3_O_10_, and SrTiO_3_. J. Chem. Thermodyn..

[23] Degueldre C., Tissot P., Lartigue H., Pouchon M. (2003). Specific heat capacity and Debye temperature of zirconia and its solid solution. Thermochim. Acta.

[24] Wan C., Motohashi Y., Shibata T., Baba S., Ishihara M., Hoshiya T. (2002). Thermal conductivity of superplastically deformed 3Y-TZP. Mater. Trans..

[25] Gugushev C., Klimm D., Langhnas F., Galazka Z., Kok D., Juda U., Uecker R. (2013). Top-seeded solution growth of SrTiO_3_ crystals and phase diagram studies in the SrO-TiO_2_ system. arXiv.

[26] Barsoum M. (1997). Fundamentals of Ceramics.

[27] Holland T.B., Anselmi-Tamburini U., Quach D.V., Tran T.B., Mukherjee A. (2012). Effects of local Joule heating during the field assisted sintering of ionic ceramics. J. Eur. Ceram. Soc..

[28] Schütze A., Jeong J.Y., Babayan S.E., Park J., Selwyn G.S., Hicks R.F. (1998). The atmospheric-pressure plasma jet: A review and comparison to other plasma sources. IEEE Trans. Plasma Sci..

[29] Hourdakis E., Simonds B.J., Zimmerman N.M. (2006). Submicron gap capacitor for measurement of breakdown voltage in air. Rev. Sci. Instrum..

[30] Sili E., Cambronne J.P., Koliatene F. (2011). Temperature dependence of electrical breakdown mechanism on the left of the Paschen minimum. IEEE Trans. Plasma Sci..

[31] Osmokrović P., Krivokapić I., Krstić S. (1994). Mechanism of electrical breakdown left of Paschen minimum. IEEE Trans. Dielectr. Electr. Insul..

[32] Hourdakis E., Bryant G.W., Zimmerman N.M. (2006). Electrical breakdown in the microscale: Testing the standard theory. J. Appl. Phys..

[33] Marder R., Estournes C., Chevallier G., Chaim R. (2015). Numerical model for sparking and plasma formation during spark plasma sintering of ceramic compacts. J. Mater. Sci..

[34] Marder R., Estournes C., Chevallier G., Chaim R. (2014). Plasma in spark plasma sintering of ceramic particle compacts. Scr. Mater..

[35] Marder R., Estournes C., Chevallier G., Chaim R. (2015). Spark and plasma in spark plasma sintering of rigid ceramic nanoparticles: A model system of YAG. J. Eur. Ceram. Soc..

[36] Holland T.B., Anselmi-Tamburini U., Quach D.V., Tran T.B., Mukherjee A. (2012). Local field strengths during early stage field assisted sintering (FAST) of dielectric materials. J. Eur. Ceram. Soc..

[37] Biesuz M., Luchi P., Quaranta A., Sglavo V.M. (2016). Theoretical and phenomenological analogies between flash sintering and dielectric breakdown in α-alumina. J. Appl. Phys..

[38] Filippov A.V., Rosner D.E. (2000). Energy transfer between an aerosol particle and gas at high temperature ratios in the Knudsen transition regime. Int. J. Heat Mass Trans..

[39] Pustovalov V.K. (2005). Theoretical study of heating of spherical nanoparticle in media by short laser pulses. Chem. Phys..

[40] Liu F., Daun K.J., Snelling D.R., Smallwood G.J. (2006). Heat conduction from a spherical nanoparticle: Status of modeling heat conduction in laser-induced incandescence. Appl. Phys. B.

[41] Merabia S., Shenogin S., Joly L., Keblinski P., Barrat J.L. (2009). Heat transfer from nanoparticles: A corresponding state physics. Proc. Natl. Acad. Sic. USA.

[42] Merabia S., Keblinski P., Joly L., Lewis L.J., Barrat J.L. (2009). Critical heat flux around strongly heated nanoparticles. Phys. Rev. E.

[43] Francis J.S.C., Cologna M., Raj R. (2012). Particle size effects in flash sintering. J. Eur. Ceram. Soc..

[44] Bykov Y.V., Egorov S.V., Eremeev A.G., Kholoptsev V.V., Plotnikov I.V., Rybakov K.I., Sorokin A.A. (2016). On the mechanism of microwave flash sintering of ceramics. Materials.

